# Extreme heat exacerbates atmospheric ozone pollution: Holistic data-based causal investigation in China

**DOI:** 10.1016/j.isci.2026.114854

**Published:** 2026-01-29

**Authors:** Ruixun Xia, Qi Qi, Jiyuan Yang, Bailiang Li, Yaoqi Li, Andrew P. Morse, Tenglong Li, Qing Mu

**Affiliations:** 1 Department of Health and Environmental Sciences, School of Science, Xi’an Jiaotong-Liverpool University, Suzhou 215123, China; 2 School of Environmental Sciences, University of Liverpool, L69 3GP Liverpool, UK; 3 School of Public Health, Brown University, Providence, RI 02903, USA; 4 Academy of Pharmacy, Xi’an Jiaotong-Liverpool University, Suzhou 215123, China

**Keywords:** Earth sciences, Atmospheric science

## Abstract

Climate change-induced extreme heat (EH) events intensify atmospheric surface ozone (O_3_) pollution, posing growing threats to public health and environment. To address the lack of rigorous empirical confirmation regarding the causal impact of EH on O_3_ despite their observed co-occurrence, this study develops a robust statistical framework to assess this relationship across China. The framework integrates three checkpoints: (1) correlational checkpoint based on linear mixed model; (2) causal checkpoint based on instrumental variable, Heckman two-step selection model, and propensity score methods with generalized bootstrap validation; and (3) directional (of causality) checkpoint based on transfer entropy. Results consistently demonstrate a statistically significant causal effect of EH on elevated O_3_ levels, with the dominant influence flowing from EH to O_3_ nationwide and across most provinces in China. These findings support integrated air quality management and climate adaptation strategies, guiding policy interventions to mitigate the compound risks of EH and O_3_ pollution.

## Highlights

• A three-step causal inference framework for extreme heat and ozone is developed
• Extreme heat is a causal driver of elevated ozone pollution across China
• Causal effect is confirmed nationally and in most Chinese provinces
• Findings necessitate integrated climate adaptation and ozone control policies

## Graphical abstract

**Figure:**
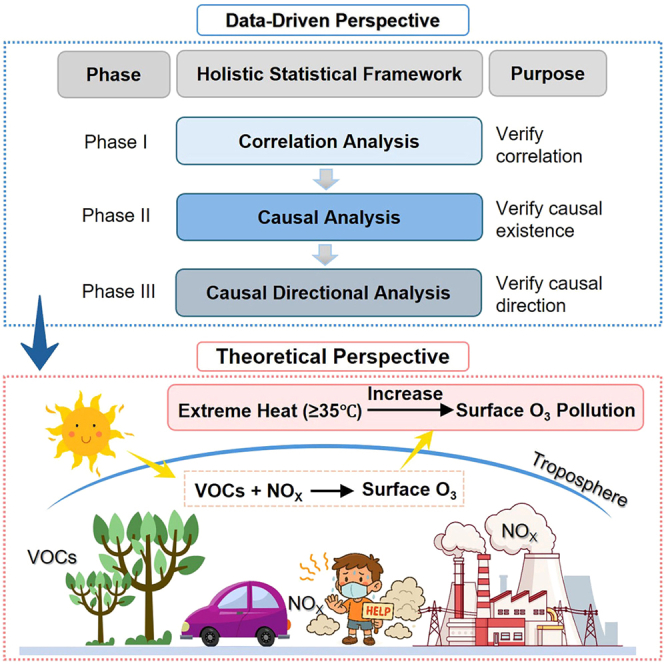

## Introduction

Global warming has led to an increase in the frequency of extreme heat (EH) events, which potentially exacerbate atmospheric surface ozone (O_3_) pollution.^1^ The combined burden poses severe risks to public health and incurs substantial economic costs globally.^2^^,^^3^^,^^4^^,^^5^ This alarming trend underscores the urgent need to disentangle the mechanisms between EH and O_3_ pollution.

The relationship between EH and O_3_ has been explored across multiple scientific fields. Chemically, elevated temperatures intensify emissions of biogenic and anthropogenic volatile organic compounds (VOCs) and nitrogen oxides (NO_X_), key precursors for O_3_,^6^^,^^7^^,^^8^^,^^9^^,^^10^ and accelerate photochemical reaction rates that promote O_3_ formation.^11^ Physically, EH is often accompanied by meteorological conditions such as strong solar radiation, weak winds, and atmospheric stagnation, which are conducive to the accumulation of O_3_ and its precursors in the boundary layer.^12^^,^^13^^,^^14^

Building on these scientific mechanisms, data-driven approaches have identified EH as a key driver of O_3_. Traditionally, multiple linear regressions have been employed to examine correlations between EH and O_3_.^15^^,^^16^^,^^17^ More recently, machine learning (ML) approaches, such as random forest,^18^^,^^19^ ridge regression,^19^ and double ML model^20^ have been applied to verify the importance of EH on O_3_. These ML methods are typically explained using SHapley Additive exPlanation analysis and are combined with process-based models to identify key contributing factors.^18^^,^^19^^,^^20^^,^^21^ However, the causal relationship between EH and O_3_ has been predominantly found based on a combination of correlational models and scientific mechanisms, rather than holistic theoretical framework in causality. The application of holistic causal inference strategy to the relationship is rather scarce, and most studies are generally constrained to a single dimension of causality. For instance, convergent cross mapping (CCM) was used to explore the causal influence of major meteorological factors on O_3_.^22^ CCM is fundamentally designed to operate on continuous variables within deterministic dynamical systems.^23^ It is not suitable for a binary variable, such as the EH indicator (yes/no). In contrast, transfer entropy (TE) is a non-parametric measure of information flow that provides insights into the direction of causality.^24^^,^^25^^,^^26^^,^^27^ It is suited for analyzing causal direction involving binary variables. However, TE alone cannot quantify the causal effect size. It is precisely these limitations that underscore the need for a holistic causal inference framework that integrates multiple checkpoints and methods.

This study investigates the causal relationship between EH and O_3_ from a holistic data-based perspective. An integrated three-step causal inference framework is adopted for analyses at both national and provincial levels in China, to provide strong evidence about causality between EH and O_3_. The framework flows from the correlational analysis to the causal analysis, and finally to the causal directional analysis to ensure statistical robustness of causality. Based on the causal inference framework, we confirm the causal effect of EH on O_3_, which underscores the urgent need for targeted air quality management and climate adaptation strategies under extreme climate.

## Results

### Framework of holistic causal inference

**Figure 1:**
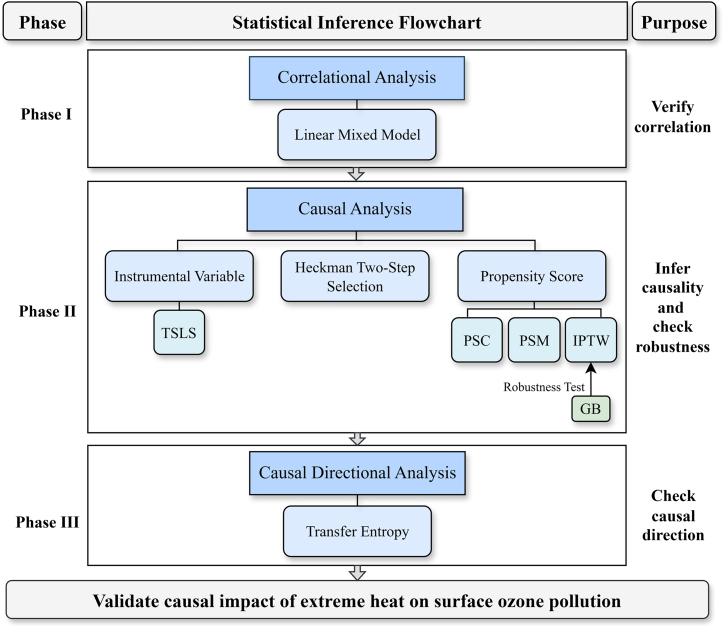
Statistical framework flowchart for assessing the causal relationship between EH and O_3_ The framework comprises three phases. Phase I involves correlation analysis using a linear mixed model (LMM) to quantify the correlation between EH and O_3_. Phase II focuses on causal inference through multiple complementary approaches, including the two-stage least squares (TSLS) model of the instrumental variable method, the Heckman two-step selection model, and propensity score analysis (propensity score calibration [PSC], propensity score matching [PSM], and inverse probability treatment weighting [IPTW]). The generalized bootstrap (GB) procedure was applied to test the robustness of IPTW. Phase III assesses the causal direction between EH and O_3_ using transfer entropy (TE).

An integrated three-step statistical framework was developed to rigorously assess the causal relationship between EH and O_3_, processing from correlational analysis, to causal analysis, and finally to determining causal directional analysis (Figure 1). For correlational analysis in phase I, a linear mixed model (LMM) was applied to account for the longitudinal/clustering nature of the dataset. For causal analysis in phase II, multiple methods were independently employed to control observables and adjust for potential confounders (so as to infer causality)^28^: two-stage least squares (TSLS) model of the instrumental variable (IV) method, Heckman two-step selection (Heckman) model, and propensity score (PS) analysis. For causal directional analysis in phase III, the TE method was used to check the causal direction.

For provincial-level analyses, the IV method and the Heckman model were not adopted, mostly because geographical homogeneity reduced the variability in altitude and made it a weak IV for addressing endogeneity/selection bias.^29^ Additionally, PSM and PSC were excluded from the model list, given prior studies have shown that small treated sample sizes can compromise covariate balance and inflate standard errors.^30^^,^^31^ Hence, we only used inverse probability treated weighting (IPTW) and generalized bootstrap (GB) for provincial-level analysis, which were relatively robust in this case.

The results across all different methods consistently suggested that EH caused an increase in O_3_ at the national level, while provincial-level analyses using robust methods confirmed such a finding in most regions, with a few exceptions. TE results further suggested a predominant causal information flow from EH to subsequent O_3_ formation at both the national and provincial levels.

### EH is positively correlated with O_3_ at national and provincial scales

**Figure 2:**
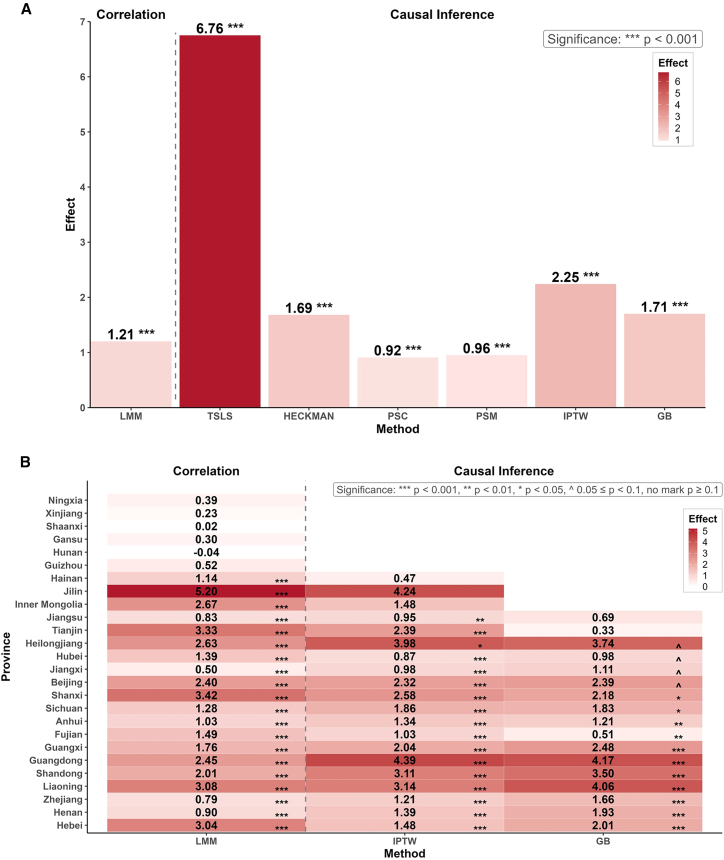
Correlational and causal analysis results between EH and O_3_ at both national and provincial levels (A) National-level results. (B) Provincial-level results for 26 provinces or municipalities (excluding Yunnan, Qinghai, and Tibet because of no EH days). Color shading indicates the magnitude of the value. The gray dashed divider separates the correlational analysis method from the causal analysis methods. Statistical significance is denoted as ∗∗∗*p* < 0.001, ∗∗*p* < 0.01, ∗*p* < 0.05, ˆ 0.05 ≦ *p* < 0.1, and no marker for *p* > 0.1. See also Tables S2–S6, which provide detailed model estimates summarized in this figure.

At the national level, the results of the LMM model yielded a significantly positive coefficient for EH (1.21, *p* < 0.001), which means that the O_3_ on EH days was 1.21 μg/m^3^ higher than that on non-EH days (Figure 2A, detailed in Table S2). This was aligned with previous research that reported positive correlations between higher temperature and O_3_ concentrations.^19^^,^^32^

Among the 29 provincial-level administrative units analyzed, EH was significantly positively correlated with O_3_ in 17 regions, including Tianjin, Jiangsu, Hebei, Henan, Zhejiang, Liaoning, Shandong, Guangdong, Guangxi, Fujian, Anhui, Sichuan, Shanxi, Beijing, Jiangxi, Hubei, and Heilongjiang (Figure 2B, detailed in Table S5). Subsequent causal analyses were conducted for national level and for those provinces with significant correlations.

### EH exerts a causal impact on O_3_ nationwide and in most provinces

Results from all methods at the national level for causal analysis supported the causal relationship between EH and O_3,_ while the causal effect estimates varied across methods (detailed in Tables S3 and S4).

The TSLS model, using altitude as a strong IV, yielded a significantly positive local average treatment effect (LATE) of 6.76 μg/m^3^ (*p* < 0.001). This LATE estimate reflects the causal effect of EH on O_3_ for the days whose EH status is influenced by altitude. Specifically, on days when altitude contributes to temperatures exceeding the 35°C threshold, exposure to EH results in an average increase of 6.76 μg/m^3^ in O_3_ after controlling for covariates.

The Heckman model similarly estimated that O_3_ would increase by 1.69 μg/m^3^ on EH days (*p* < 0.001), adjusting for selection bias. To assess the validity of the PS-based analyses, the overlap of PS distributions, balance before and after matching, and standardized mean differences were examined at the national level (Figures S2–S4). Among PS applications, both PSC and PSM estimated the average treatment effect on the treated (ATT), yielding increases of 0.92 and 0.96 μg/m^3^, respectively (*p* < 0.001). The ATT represents the average increase in O_3_ on days with EH compared with what those same days would be expected to have under similar meteorological and environmental conditions but without EH. In contrast, the IPTW method estimated the average treatment effect (ATE) to be 2.25 μg/m^3^ (*p* < 0.001), representing the expected change in O_3_ if all days were counterfactually exposed to EH compared with none. To test the robustness of IPTW, the conservative GB procedure was conducted and led to a significant ATE estimate of 1.71 μg/m^3^ (*p* < 0.001) (Figure S5).

Compared with previous studies solely relying on ML approaches (e.g., double ML) or empirical causality methods (e.g., CCM),^20^^,^^22^ our multi-methods approach provided more reliable and stronger evidence. Although some studies reported the existence of causality between EH and O_3_, they were mostly built on predictive or correlational frameworks with limited capacity for causal inference.^22^ In contrast, our results demonstrated that even after correcting for confounding and bias through multiple complementary methods, the causal relationship remained robust at the national level.

Despite its strengths, results became more divergent when this multi-method approach was applied at the provincial level. For most individual provinces, IPTW and GB yielded significantly consistent results, including Hebei, Henan, Zhejiang, Liaoning, Shandong, Guangdong, Guangxi, Fujian, Anhui, Sichuan, and Shanxi (*p* < 0.05, Figure 2B). This confirmed the robustness of the causal relationship between EH and O_3_ in these provinces. Notably, although the GB-based results for Beijing (*p* = 0.06), Jiangxi (*p* = 0.08), Hubei (*p* = 0.07), and Heilongjiang (*p* = 0.06) slightly exceed 0.05, those *p*-values fall within the range of marginal significance. These results, in conjunction with the strongly significant IPTW estimates in the same provinces, indicated the potential existence of a causal relationship between EH and O_3_ in these provinces. The estimated ATE in Tianjin and Jiangsu were found to be statistically significant using the IPTW but insignificant using the GB because the GB procedure tended to provide more conservative significance assessments. Specifically, the gap in Tianjin may be due to high resampling variability from limited sample size and fewer EH days, and the gap in Jiangsu may be due to differences in the geographical environment among sites.^33^ Based on this, a causal effect of EH on O_3_ in Tianjin and Jiangsu was not confirmed by the most conservative robustness check (GB procedure) in this study, and thus causality in these two provinces should be made with caution (detailed in Table S6).

### EH consistently drives subsequent increases in O_3_

**Figure 3:**
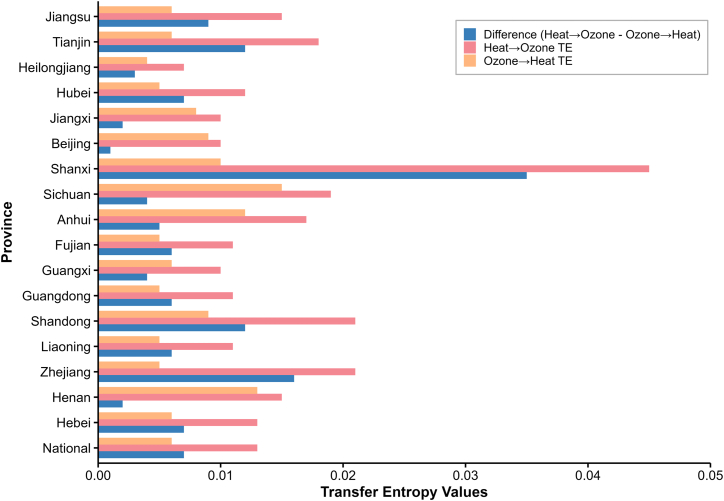
TE analysis between EH and O_3_ The bar chart presents the TE values for two directional relationships, along with the difference. The Heat to O_3_ TE (pink bars) and O_3_ to Heat TE (orange bars) represent the estimated information flow from EH to O_3_ and from O_3_ to EH, respectively. The Difference (blue bars) is calculated by subtracting two calculated entropies. Provinces where TE from heat to O_3_ exceeds the reverse direction suggest that EH has a stronger informational influence on subsequent O_3_ variation, implying potential causality direction. The “National” bar represents the aggregated national-level TE. See also Table S7.

TE was applied to assess the temporal precedence between EH and O_3_, thereby deciding the directionality of their causal relationship. At the national level, the estimated TE from EH to O_3_ was 0.013 (*p* < 0.001), which was more than twice that in the reverse direction (TE = 0.006 from O_3_ to EH, *p* < 0.001). The positive difference of the two directions (TE difference = 0.007, *p* < 0.001) indicated a predominant causal influence of EH on subsequent O_3_ formation nationwide (Figure 3; Table S7).

At the provincial level, among the provinces with confirmed causality, TE values from EH to O_3_ remained consistently higher than TE values from O_3_ to EH (Figure 3; Table S7). This agreed with the national result that EH caused an increase in O_3_ rather than the other way around. Regarding the special case (insignificant GB results) in Tianjin and Jiangsu, both had significantly higher TE values from EH to O_3_ compared with TE values from the opposite direction, which again supported the causal direction from the other provinces (Tianjin: 0.018 from EH to O_3_ > 0.006 from O_3_ to EH, *p* < 0.001; Jiangsu: 0.015 from EH to O_3_ > 0.006 from O_3_ to EH, *p* < 0.001). This highlights the complementary nature of different methods within our framework: while the IPTW/GB trio aims to quantify a robust causal effect, TE provides independent evidence regarding the direction of influence.

The TE-based evidence reinforced the causal analysis that EH substantially increased O_3_ at both national and provincial levels.

## Discussion

### Principal findings

This study establishes a robust three-step causal inference framework and reported that EH is a statistically significant causal driver of elevated O_3_ levels at the national level and in most Chinese provinces. Our findings were consistent with scientific theoretical analyses that showed EH catalyzed O_3_ formation.^1^^,^^11^^,^^14^ The work moves beyond correlational and predictive studies by applying multiple statistical methods with different assumptions to triangulate evidence. The consistent results across the methods strengthen the credibility of our conclusion and indicate that the association is driven by confounding.

### Policy implications

The variation in the magnitude of the causal effect estimates across different methods offers nuanced insights rather than indicating inconsistency. From a practical perspective, the LATE from the IV method, the ATT from PSM and PSC, and the ATE from IPTW each answer a different policy-relevant question. The LATE identifies geographic vulnerability hotspots where the impact of EH is most acute, the ATT quantifies the actual public health burden of observed EH events, and the ATE forecasts the population-wide benefit of large-scale climate adaptation strategies. The effects offer complementary evidence for crafting targeted to broad-scale interventions. Notably, the convergence in their statistical significance is the most critical finding, underscoring that EH exerts a reliable causal influence on O_3_, regardless of the specific subpopulation or estimand considered. Therefore, our findings show that heat adaptation and air-quality management must be addressed together.

### Limitations of the study

Despite the robustly holistic causal framework employed, several limitations should be acknowledged. First, our causal inference methods rely on assumptions, and unmeasured confounding such as atmospheric chemistry or emission dynamics cannot be completely excluded. Second, some provincial-level analyses may be limited, particularly in regions with few EH events (e.g., Tianjin), which may undermine the overlap assumption for PS methods and result in biases.

### Future directions

These limitations naturally chart a course for future research. To address potential unmeasured confounding and model assumptions, future studies should integrate emerging ML techniques with traditional causal inference frameworks to better handle high-dimensional confounders and complex, non-linear relationships.^34^ Furthermore, the provincial heterogeneity and limited statistical power in certain regions highlight the need to investigate the drivers of spatial variation, such as regional differences in ozone chemical regimes (e.g., VOC-limited vs. NO_X_-limited), industrial structures, and land-use patterns, which is essential for developing tailored control strategies. Extending this holistic causal investigation to other global regions, such as Europe and North America,^35^ will further test the generalizability of our findings, help overcome data limitations in specific areas, and inform global environmental policy.

## Resource availability

### Lead contact

Requests for further information and resources should be directed to and will be fulfilled by the lead contact, Qing Mu (qing.mu@xjtlu.edu.cn).

### Materials availability

This study did not generate new unique reagents.

### Data and code availability

• The cleaned dataset in this study is available in the Zenodo repository at https://doi.org/10.5281/zenodo.17148981.
• The analysis codes are developed using R (version 4.4) and are available in the same Zenodo repository at https://doi.org/10.5281/zenodo.17148981.
• Upon reasonable request, the lead contact can provide additional analyses.

## Acknowledgments

This study was funded by the Basic Research Program of Jiangsu (BK20250467), the Research Development Fund (No. RDF23-01-118) at 10.13039/501100006683Xi’an Jiaotong-Liverpool University, and the Jiangsu Province Young Scientific and Technological Talents Promotion Plan (No. JSTJ-2024-436).

## Author contributions

Conceptualization, Q.M.; methodology, T.L.; formal analysis, R.X., Q.Q., T.L., and Q.M.; data curation, R.X.; visualization, R.X. and Q.Q.; investigation, R.X. and Q.Q.; writing – original draft, R.X.; writing – review & editing, R.X., Q.Q., J.Y., B.L., Y.L., A.P.M., T.L., and Q.M.; supervision, T.L. and Q.M.; resources, Q.M.; project administration, Q.M.; funding acquisition, Q.M.

## Declaration of interests

The authors declare no competing interests.

## STAR★Methods

### Key resources table

**Table:** 

REAGENT or RESOURCE	SOURCE	IDENTIFIER
**Deposited data**		

Air quality monitoring measurements	China National Environmental Monitoring Center	https://air.cnemc.cn:18007/
Meteorological dataset	National Oceanic and Atmospheric Administration	https://www.ncei.noaa.gov/
Cleaned dataset for analysis	This paper	https://doi.org/10.5281/zenodo.17148981

**Software and algorithms**		

Draw.io	Web-based Diagramming Platform	https://www.drawio.com/
R version 4.4	R Software	https://www.r-project.org/
Linear Mixed Model	Bates et al.^36^	https://doi.org/10.18637/jss.v067.i01
Instrumental Variable	Hogan and Lanchaster^37^	https://doi.org/10.1191/0962280204sm351ra
Heckman Two-step Selection Model	Heckman^38^	https://doi.org/10.2307/1909757
Propensity Score Analysis	Rosenbaum and Rubin^39^	https://doi.org/10.1093/biomet/70.1.41
Generalized Bootstrap	Li and Lawson^33^	https://doi.org/10.1080/00273171.2023.2254541
Code	This paper	https://doi.org/10.5281/zenodo.17148981

### Method details

#### Data description

Observed hourly O_3_ concentration data and hourly meteorological observations from 2014 to 2023 were obtained from the China Environmental Monitoring Centre and the National Climate Data Centre under the U.S. National Oceanic and Atmospheric Administration, respectively (Methods S1). The daily maximum 8-hour average (MDA8) O_3_ and the daily maximum temperature were computed. Following the guidance about EH days by the China Meteorological Administration and previous studies,^40^ EH days were defined as days with daily maximum temperatures beyond 35°C. The EH days were implemented through a binary variable (1 for EH days, 0 for non-EH days). This binary definition is not only policy-relevant but is also methodologically requisite for establishing a clear treatment-control structure, which is fundamental to the causal inference framework employed in this study.

To maintain consistency throughout the dataset, data fusion and preprocessing were performed (Methods S1). The data collocation process involved merging the daily meteorological and air-quality records based on city and site identifiers to ensure spatial-temporal alignment. As part of a rigorous quality control procedure, sporadic missing values were addressed via linear interpolation, while sites with substantial data gaps or uncertain matches were excluded. The daily averages of other meteorological factors, including dew point temperature, sea level pressure, wind direction, wind speed rate, sky condition total coverage, and liquid precipitation were computed during the data preprocessing stage (Table S1). The final dataset included 97 sites across 29 provincial-level units from 2014 to 2023 in China (Figure S1). The preprocessed dataset was first utilized to conduct national-level analysis and then grouped into 29 subsets based on the site location for provincial-level analysis.

#### Statistical methods within a holistic framework

In Phase I (Figure 1), the LMM model (Methods S2) incorporated fixed effects for EH on O_3_ with random intercepts accounting for unobserved site-level heterogeneity. The expression of the model is given by Equation 1:

(Equation 1) $$\begin{array}{c} Y_{ij}=\beta_{0}+\beta_{1}D_{ij}+\beta X_{ij}+b_{j}+\epsilon_{ij} \end{array}$$

where *Y*_*ij*_ is the MDA8 O_3_ for day *i* in region *j*. *D*_*ij*_ is EH events. *X*_*ij*_ is a vector of additional fixed effect covariates, including dew point temperature, sea level pressure, wind direction, wind speed rate, sky condition total coverage, and liquid precipitation. *β* is the corresponding vector of fixed effect coefficients. *b*_*j*_ is the random intercept for region *j*, capturing unobserved regional heterogeneity. *ϵ*_*ij*_ is the residual error term, assumed to be independent of *b*_*j*_. The fixed effects estimate the average relationship across all regions, while the random effects account for deviations specific to each region. This hierarchical modelling structure improves statistical efficiency and yields more reliable standard errors in the presence of within-group correlation. Notably, in within-provincial analysis, Beijing, Sichuan, Tianjin, and Ningxia each have only one site; thus, we used the linear model (LM) without random effects to explore the correlation in these areas. A statistically significant correlation between EH and O_3_ was considered as a prerequisite for potential causal links between the two. Hence, the correlation analysis was carried out at the national and provincial levels before inferring causality.

In Phase II (Figure 1), the aim was to infer the existence of causality and estimate the causal effect of EH on O_3_. The EH was considered the treatment, O_3_ concentration was considered the outcome, and meteorological factors were considered covariates. For national-level analysis, the IV method, the Heckman model, and the PS analysis were adopted. Specifically, the IV method was implemented using a TSLS model (Methods S3), with altitude serving as the IV to address endogeneity of EH.^37^^,^^41^^,^^42^^,^^43^ The model can be expressed as two stages. In the first stage, we estimated a predictive model for EH using altitude as an instrument and other exogenous controls, Equation 2:

(Equation 2) $$\begin{array}{c} X_{ij}=\pi_{0}+\pi IV_{ij}+\theta W_{ij}+u_{ij} \end{array}$$

where *X*_*ij*_ is EH for day *i* in site *j*, *IV*_*ij*_ is altitude, *w*_*ij*_ includes other exogenous meteorological covariates. *u*_*ij*_ is the error term. This stage yields predicted values ${\hat{X}}_{ij}$, representing the component of EH variation attributable to exogenous variation in altitude. In the second stage, we then modelled O_3_ concentration using the predicted values ${\hat{X}}_{ij}$ as the explanatory variable in an ordinary least square (OLS) model, Equation 3:

(Equation 3) $$\begin{array}{c} Y_{ij}=\beta_{0}+\delta {\hat{X}}_{ij}+{\beta X}_{ij}+\epsilon_{ij} \end{array}$$

where *Y*_*ij*_ is the MDA8 O_3_, *X*_*ij*_ includes other meteorological covariates. Similarly, *ϵ*_*ij*_ is the error term. The coefficient *δ* captures the causal effect of EH on O_3_ levels, adjusted for endogeneity.

The Heckman model (Methods S4) aimed to correct sample selection bias due to data representativeness.^38^ In the first stage, we estimated the probability of EH occurrence using a probit model Equation 4:

(Equation 4) $$\begin{array}{c} D_{i}^{*}=Z_{i}\gamma +u_{i} \end{array}$$

where *D*_*i*_ is a binary indicator for EH on day *i*, and *Z*_*i*_ is a vector of covariates that determine the likelihood of EH, including altitude and meteorological factors. The error term *u*_*i*_ follows a standard normal distribution. This model yields estimates of *γ*, which we used to compute the Inverse Mills Ratio (IMR). The IMR captures the conditional probability of being observed in the treated group and adjusts for non-random selection in the second stage. In the second stage, we estimated the causal impact of EH on O_3_ using a LMM that includes the IMR as a covariate, Equation 5:

(Equation 5) $$\begin{array}{c} Y_{ij}=\beta_{0}+\delta D_{ij}+X_{ij}\beta +\rho \lambda_{ij}+b_{j}+\epsilon_{ij} \end{array}$$

*Y*_*ij*_ is the MDA8 O_3_ for day *i* in site *j*. *D*_*ij*_ is the treatment indicator for EH. *X*_*ij*_ is a vector of covariates including meteorological variables. *λ*_*ij*_ represents the IMR estimated from the first stage. *b*_*j*_ is a random intercept that accounts for unobserved heterogeneity across sites and *ϵ*_*ij*_ is the error term. The coefficient *δ* captures the adjusted causal effect of EH on O_3_. The term *ρ* indicates the extent of selection bias and tests whether correction is necessary. The inclusion of a random effect *b*_*j*_ controls for correlation within spatial clusters.

Subsequently, PS analysis (Methods S5), including propensity score calibration (PSC), propensity score matching (PSM), and inverse probability treated weighting (IPTW), was utilized to imitate the conditions of randomized controlled trials for reducing potential confounding biases in observational studies based on the assumption of unconfoundedness.^39^ In our analysis, we calculated propensity scores as the probability of being exposed to EH. The scores were estimated via logistic regression, Equation 6:

(Equation 6) $$\begin{array}{c} e\left(X_{i}\right)=P\left({\text{EH}}_{i}=1|X_{i}\right) \end{array}$$

where EH_*i*_ is day *i* in EH (the treatment) group or not, and *e*(*X*_*i*_) is the probability that day *i* is EH day. Furthermore, the GB (Methods S6) was a conservative procedure for correcting the underestimated standard error associated with IPTW,^33^ which means statistical significance under GB has higher credibility and thus can be used to test the robustness of IPTW.

In Phase III (Figure 1), TE (Methods S7) was applied additionally to identify the causal direction, provided that causality could be established in Phase II. TE quantified the information flow between two variables using conditional probability distributions, allowing for the detection of nonlinear relationships and the direction of influence.^44^ The source variable (X) is EH, and the target variable (Y) is O_3_. We depend on the difference between the conditional entropy of EH and O_3_ to differentiate their sequences of occurrence. Here is the formula in terms of the temporal sequence:

(Equation 7) $$\begin{array}{c} T_{X\to Y}=H\left(y_{t+1}|y_{t}^{L}\right)-H\left(y_{t+1}|y_{t}^{L},x_{t}^{K}\right) \end{array}$$

where $y_{t}^{L}=\left\{y_{t},y_{t-1},\ldots ,y_{t-L+1}\right\}$ denotes the past *L* values of *Y*, $x_{t}^{K}=\left\{x_{t},x_{t-1},\ldots ,x_{t-K+1}\right\}$ denotes the past *K* values of *X*. $H\left(y_{t+1}|y_{t}^{L}\right)$ represents the uncertainty in predicting *y*_*t*+1_ using only the past information of *Y*. $H\left(y_{t+1}|y_{t}^{L},x_{t}^{K}\right)$ represent the uncertainty in predicting *y*_*t*+1_ using both the past information of *Y* and *X*. If $H\left(y_{t+1}|y_{t}^{L},x_{t}^{K}\right)$ is significantly smaller than $H\left(y_{t+1}|y_{t}^{L}\right)$, then $T_{X\to Y}>0$, indicating that *X* provides additional predictive information about *Y*. TE was calculated for two directions: from EH to O_3_, and from O_3_ to EH. Causal direction was determined by comparing the TE values associated with the two directions. If TE from EH to O_3_ exceeded that from O_3_ to EH, EH was considered to be the cause of O_3_.

### Quantification and statistical analysis

All statistical analyses and data visualizations presented in this study were conducted using R (version 4.4) within the RStudio integrated development environment. The conceptual flowchart illustrating the holistic causal inference framework (Figure 1) was created using the web-based diagramming platform draw.io.
