# Supplementary material for: Extreme heat exacerbates atmospheric ozone pollution: Holistic data-based causal investigation in China
Source: iScience. 2026 Jan 29;29(3):114854. doi: 10.1016/j.isci.2026.114854 (PMC12925047; doi:10.1016/j.isci.2026.114854)
Supplement: Document S1. Figures S1–S5, Tables S1–S7, and Methods S1–S7 [file mmc1.pdf]

iScience, Volume *29*

Supplemental information

Extreme heat exacerbates atmospheric ozone pollution: Holistic data-based causal investigation in China

Ruixun Xia, Qi Qi, Jiyuan Yang, Bailiang Li, Yaoqi Li, Andrew P. Morse, Tenglong Li, and Qing Mu

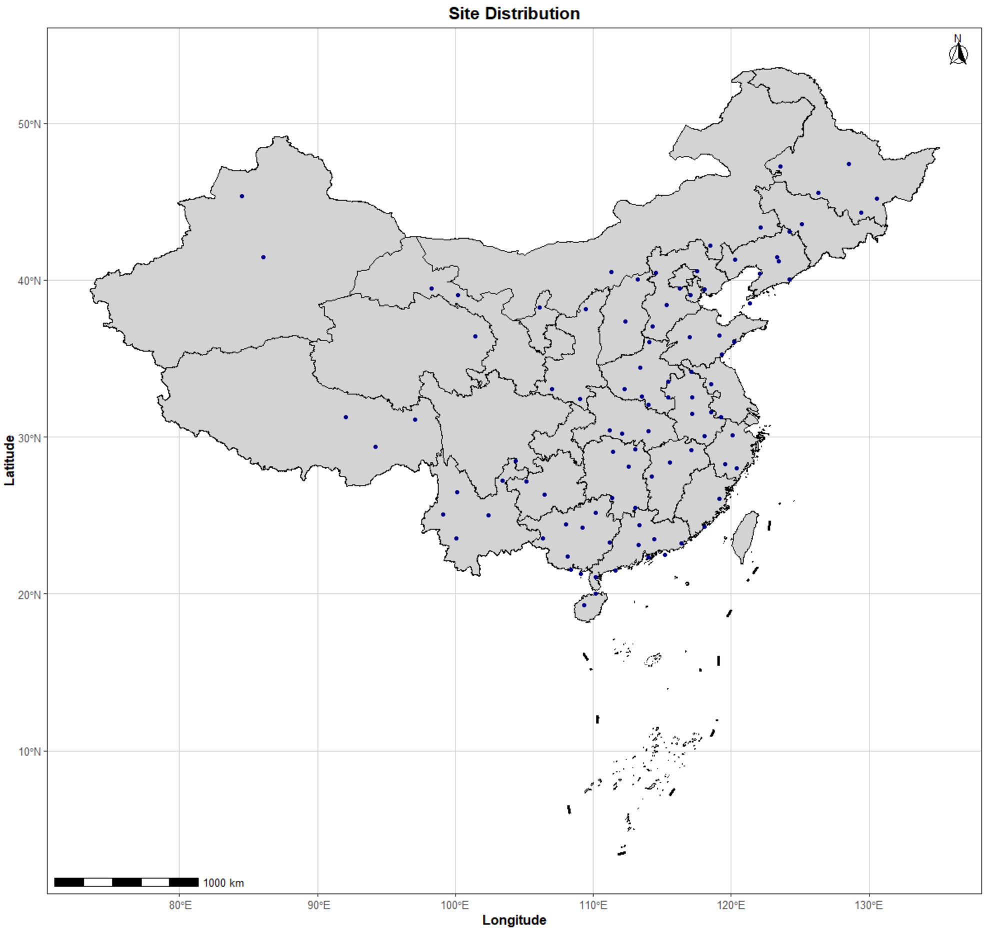

Figure S1. Monitoring Sites Distribution. There are 97 monitoring sites across 29 provinces and municipalities in mainland China.

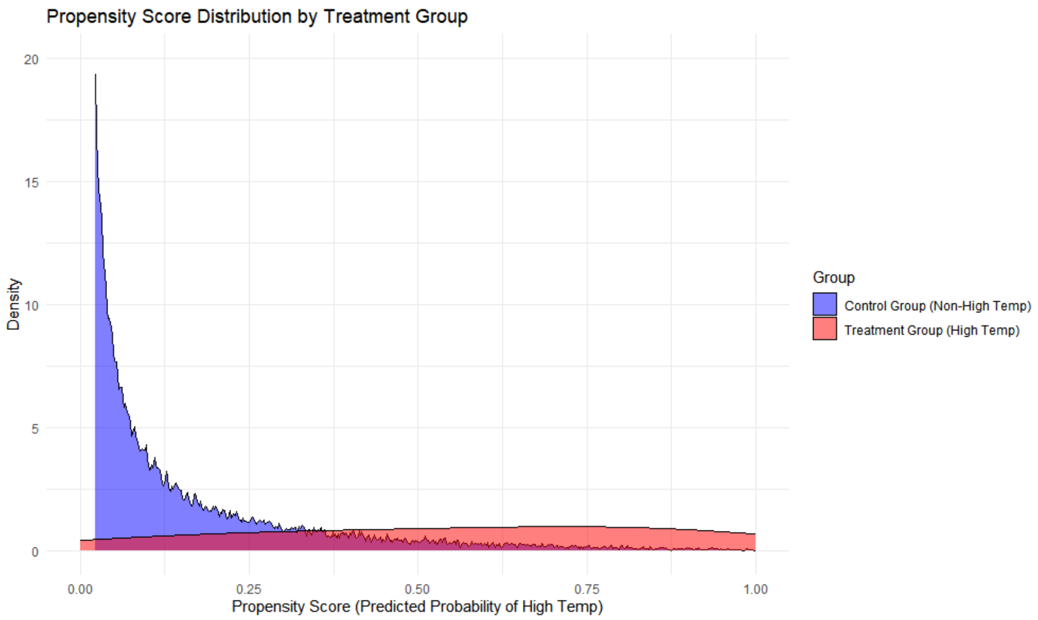

Figure S2. Propensity Score Density Distributions of EH Group (Treatment Group) and Non-EH Group (Control Group) at the National Level. Notably, researchers generally begin by examining the PS distribution using histograms or density plots to ensure sufficient overlap between groups before applying PSM. According to the figure, we found some overlap between the two groups, suggesting PSM is possible. There was a quantity of treated observations with high PS while only few control observations with high PS. Given the observed imbalance, 2:1 NN matching criteria was used, with replacement, to improve matching quality and reduce bias.[1,2]

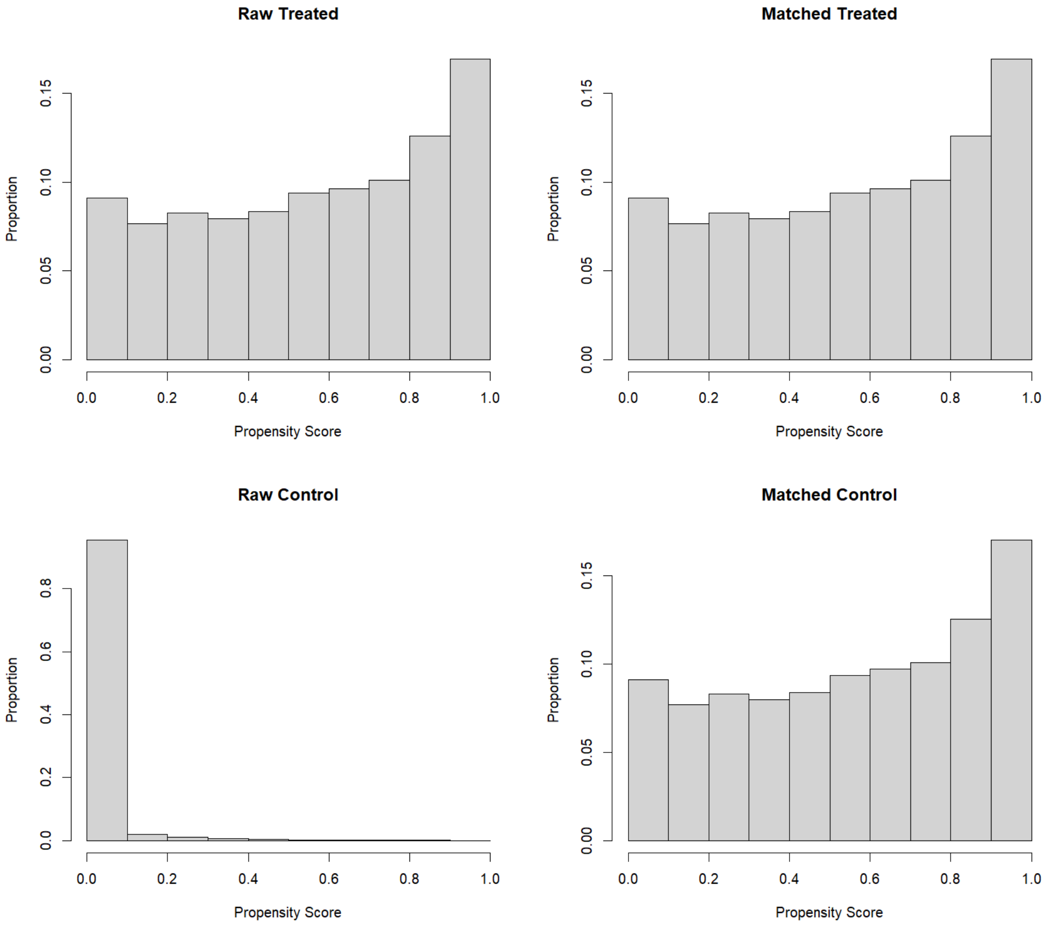

Figure S3. Propensity Score Distribution before and after PSM at the National Level. The histograms showed improved balance in covariate distributions after matching.

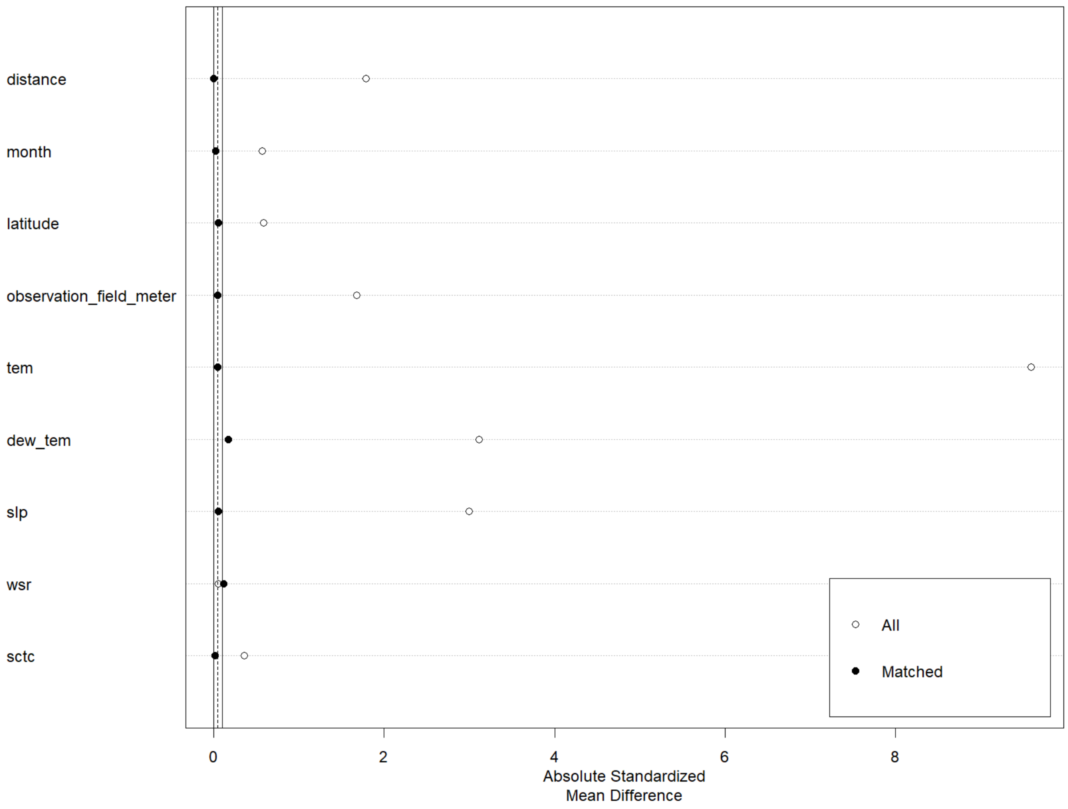

Figure S4. Absolute Standardized Mean Difference (SMD) between Two Groups before and after Matching at the National Level. After matching, the adjusted SMD values fell below the accepted threshold of 0.1. The improved balance enhanced the comparability between the groups and allowed for a more reliable causal inference.

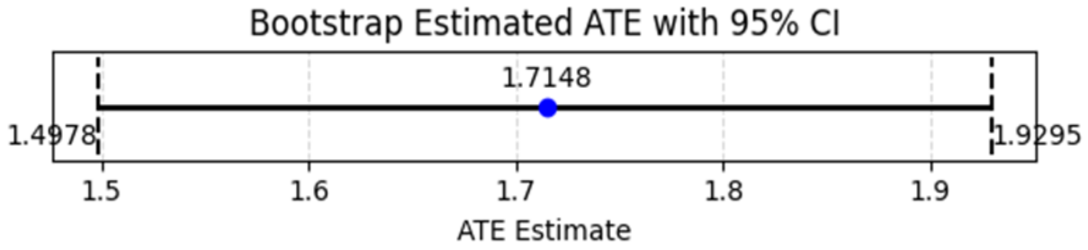

Figure S5. The Estimated Average Treatment Effect by GB Procedure at the National Level. The 95% confidence interval (CI) for the effect estimate ranged from 1.4978 to 1.9295, indicating a statistically significant and robust effect.

Table S1. Comprehensive Variable Specifications for Statistical Modelling.

Variable	Description
O_3_8h_max	The daily maximum 8-hour average of O_3 concentrations (MDA8).
max_tem_35	Indicator for whether the daily maximum air temperature reaches or exceeds 35°C (1 for yes, 0 for no).
tem_max	The daily maximum air temperature.
dew_tem	The dew point temperature, which is the temperature at which air must be cooled, at constant pressure and water vapor content, for saturation to occur.
slp	Sea level pressure, the atmospheric pressure adjusted to sea level.
wd	Wind direction, defined as the angle measured clockwise from true north to the direction from which the wind is blowing.
wsr	Wind speed rate, indicating the horizontal velocity of air at a fixed point.
sctc	Sky condition total coverage, the fraction of the sky covered by clouds or other obscuring phenomena.
lpd	The depth of liquid precipitation.
latitude	The geographic latitude of the monitoring sites.
observation_field_meter	The altitude of the meteorological observation site.

Table S2. Statistical Results of LMM at the National Level.

Predictors	Estimates	SE	VIF
(Intercept)	14.91***	0.12	
max_tem_35	1.21***	0.03	1.07
dew_tem	0.57***	0.01	3.10
slp	-1.4***	0.01	3.06
wd	-0.23***	0.01	1.03
wsr	0.31***	0.01	1.07
sctc	-0.78***	0.01	1.05
lpd	-0.32***	0.01	1.04
**Random effects**			
σ^2	9.05		
Site τ_{00}	1.32		
ICC	0.13		
Site N	97		
Observations	304713		
Marginal R^2	0.282		
Conditional R^2	0.374		
Likelihood ratio test (p-value)	<0.001		

Notes. *** represents p values < 0.001.

Table S3. Statistical Results of TSLS and Heckman at the National Level.

	TSLS		Heckman's Two-step	
**Predictors**	**Estimates**	**SE**	**Estimates**	**SE**
(Intercept)	14.72***	0.02	35.76***	0.50
max_tem_35	6.76***	0.49	1.69***	0.03
dew_tem	0.04**	0.02	-3.99***	0.09
slp	-1.37***	0.02	3.86***	0.10
wd	-0.22***	0.01	-0.61***	0.01
wsr	0.18***	0.01	0.49***	0.01
sctc	-0.61***	0.01	0.76***	0.03
lpd	-0.29***	0.01	0.28***	0.01
IMR			-5.75***	0.11
R^2	0.17			
Adjust R^2	0.17			
Weak instrument test (p-value)	<0.001			
Wu-Hausman test (p-value)	<0.001			
**Random effects**	No		Yes	
σ^2			8.97	
Site τ_{00}			7.73	
ICC			0.46	
Site N			97	
Observations			304713	
Marginal R^2			0.349	
Conditional R^2			0.651	
Likelihood ratio test (p-value)			<0.001	

Notes. *** &** represent p values <0.001 and <0.05 respectively.

Table S4. Statistical Results of PS Methods at the National Level.

	PSC		IPTW		PSM	
**Predictors**	**Estimates**	**SE**	**Estimates**	**SE**	**Estimates**	**SE**
(Intercept)	14.73***	0.09	14.92***	0.12	18.06***	0.26
max_tem_35	0.92***	0.05	2.25***	0.04	0.96***	0.04
dew_tem			0.58***	0.01		
slp			-1.38***	0.01		
wd			-0.21***	0.01		
wsr			0.32***	0.01		
sctc			-0.82***	0.01		
lpd			-0.24***	0.00		
propensity score	4.95***	0.07				
**Random effects**	Yes		Yes		Yes	
σ^2	12.09		8.64		5.86	
Site τ_{00}	0.8		1.35		5.59	
ICC	0.06		0.14		0.49	
Site N	97		97		90	
Observations	304713		304713		19542	
Marginal R^2	0.050		0.304		0.019	
Conditional R^2	0.109		0.398		0.498	
Likelihood ratio test	<0.001		<0.001		<0.001	

Notes. *** represents p values <0.001.

Table S5. Results of Correlation by Province.

Province	Effect	SE	P value	Model type
Yunnan				Skipped
Inner Mongolia	2.669	0.290	0.000***	LMM
Beijing	2.395	0.266	0.000***	LM
Jilin	5.204	0.665	0.000***	LMM
Sichuan	1.277	0.213	0.000***	LM
Tianjin	3.327	0.260	0.000***	LM
Ningxia	0.389	0.371	0.295	LM
Anhui	1.032	0.118	0.000***	LMM
Shandong	2.005	0.175	0.000***	LMM
Shanxi	3.415	0.298	0.000***	LMM
Guangdong	2.451	0.085	0.000***	LMM
Guangxi	1.758	0.079	0.000***	LMM
Xinjiang	0.232	0.136	0.089	LMM
Jiangsu	0.834	0.138	0.000***	LMM
Jiangxi	0.495	0.136	0.000***	LMM
Hebei	3.036	0.184	0.000***	LMM
Henan	0.900	0.125	0.000***	LMM
Zhejiang	0.794	0.138	0.000***	LMM
Hainan	1.143	0.218	0.000***	LMM
Hubei	1.386	0.141	0.000***	LMM
Hunan	-0.042	0.121	0.730	LMM
Gansu	0.303	0.178	0.089	LMM
Fujian	1.485	0.118	0.000***	LMM
Tibet				Skipped
Guizhou	0.523	0.656	0.425	LMM
Liaoning	3.082	0.253	0.000***	LMM
Shaanxi	0.021	0.144	0.886	LMM
Qinghai				Skipped
Heilongjiang	2.633	0.585	0.000***	LMM

Notes. Statistical significance is denoted as *** $p < 0.001$, ** $p < 0.01$, * $p < 0.05$, ^ $0.05 \leqq p < 0.1$ and no marker for $p \geqq 0.1$. Yunnan, Qinghai, and Tibet were excluded because of the absence of EH days. Beijing, Sichuan, Tianjin, and Ningxia each have only one site, so we used LM for these areas to explore the correlations.

Table S6. Results of IPTW and GB by Province.

	IPTW			GB		
**Province**	**Effect**	**SE**	**P value**	**Effect**	**SE**	**P value**
Liaoning	3.139	0.427	0.000***	4.057	0.970	0.004***
Zhejiang	1.212	0.182	0.000***	1.658	0.356	0.000***
Hebei	1.477	0.397	0.000***	2.013	0.641	0.000***
Beijing	2.319	0.572	0.000***	2.388	1.241	0.066^
Guangxi	2.036	0.119	0.000***	2.482	0.388	0.000***
Jiangsu	0.954	0.318	0.003**	0.687	0.535	0.198
Jiangxi	0.982	0.216	0.000***	1.107	0.633	0.082^
Fujian	1.026	0.157	0.000***	0.506	0.211	0.006**
Anhui	1.337	0.180	0.000***	1.211	0.430	0.006**
Shanxi	2.578	0.497	0.000***	2.184	1.062	0.035*
Tianjin	2.385	0.593	0.000***	0.328	1.090	0.930
Henan	1.388	0.154	0.000***	1.931	0.454	0.000***
Sichuan	1.858	0.436	0.000***	1.832	0.444	0.036*
Hubei	0.867	0.151	0.000***	0.980	0.544	0.074^
Guangdong	4.392	0.077	0.000***	4.170	0.289	0.000***
Shandong	3.106	0.402	0.000***	3.496	0.517	0.000***
Heilongjiang	3.982	1.990	0.045*	3.740	0.951	0.056^
Inner Mongolia	1.480	0.912	0.105			
Jilin	4.238	5.241	0.419			
Xinjiang	0.163	0.313	0.603			
Hainan	0.470	0.438	0.283			
Gansu	0.030	0.740	0.968			

Notes. Statistical significance is denoted as *** $p < 0.001$, ** $p < 0.01$, * $p < 0.05$, ^ $0.05 \leqq p < 0.1$ and no marker for $p \geqq 0.1$.

Table S7. Results of TE by Province and Nation.

Province	Heat to O_3	p-value	O_3 to Heat	p-value	Direction
National	0.013	0.00***	0.006	0.00***	Heat to O_3
Liaoning	0.011	0.00***	0.005	0.00***	Heat to O_3
Zhejiang	0.021	0.00***	0.005	0.00***	Heat to O_3
Hebei	0.013	0.00***	0.006	0.00***	Heat to O_3
Beijing	0.010	0.00***	0.009	0.00***	Heat to O_3
Guangxi	0.010	0.00***	0.006	0.00***	Heat to O_3
Jiangsu	0.015	0.00***	0.006	0.00***	Heat to O_3
Jiangxi	0.010	0.00***	0.008	0.00***	Heat to O_3
Fujian	0.011	0.00***	0.005	0.00***	Heat to O_3
Anhui	0.017	0.00***	0.012	0.00***	Heat to O_3
Shanxi	0.045	0.00***	0.010	0.00***	Heat to O_3
Tianjin	0.018	0.00***	0.006	0.02*	Heat to O_3
Henan	0.015	0.00***	0.013	0.00***	Heat to O_3
Sichuan	0.019	0.00***	0.015	0.00***	Heat to O_3
Hubei	0.012	0.00***	0.005	0.00***	Heat to O_3
Guangdong	0.011	0.00***	0.005	0.00***	Heat to O_3
Shandong	0.021	0.00***	0.009	0.00***	Heat to O_3
Heilongjiang	0.007	0.00***	0.004	0.00***	Heat to O_3

Notes. *** &* represent p values <0.001 and <0.05 respectively. This table summarizes the bidirectional transfer entropy analysis between EH and O_3 at both the national and provincial levels.

Methods S1. Notes for Data Source and Data Preprocessing.

The hourly meteorological data and hourly air quality data were originally sourced from the National Climatic Data Centre (NCDC) and the China National Environmental Monitoring Centre (CNEMC). Scholar Xiaolei Wang collected and arranged these data for research purposes; thus, data are also available from: https://quotsoft.net/air/. In this study, we used data from 2014 to 2023. The starting year 2014 was chosen because the national air quality monitoring network in China has provided reliable observations only since then, while 2023 marked the last complete year available when the data extraction was carried out in 2024.

For meteorological data, hourly meteorological data were selected and converted into CSV format, and all monitoring site records were aggregated by year into a single dataset for each year from 0:00 on January 1st to 23:00 on December 31st. Based on site id, the dataset was then matched with the site information table from the China Surface Meteorological Observation Database. Basic information for stations, including latitude, longitude, and altitude, was added to the dataset. Hourly meteorological data were converted to daily data, and daily maximum temperature was calculated by grouping hourly observations by date and extracting the highest value for each 24-hour period. Annual meteorological datasets were finally combined into one large dataset from 2014 to 2023.

For air quality data, hourly air quality data were compiled into yearly datasets, each dataset covering 0:00 on January 1st to 23:00 on December 31st for all cities in that year. Based on cities, the corresponding province information was added to the datasets. Hourly air quality data were converted to daily data, and the daily maximum 8-hour moving average (MDA8) ozone (O_3) data were extracted from data sets because 24-hour MDA8 O_3 was already contained in the data sets. Similarly, annual air quality data sets were finally combined into one large data set from 2014 to 2023.

Considering that meteorological data and air quality data come from two data sources, we had to merge two data sets by city and site names, regions where meteorological data and air quality data could not be matched were excluded, sporadic missing data were filled using linear interpolation[3], while sites with continuous missing data were excluded, resulting in a final data set comprising 97 monitoring sites across 29 provinces and municipalities in mainland China from 2014 to 2023.

Because the O_3 data exhibited right skewness, violating the normality assumption required for statistical modelling, a Box-Cox transformation was applied to approximate a normal distribution Eq.S1.

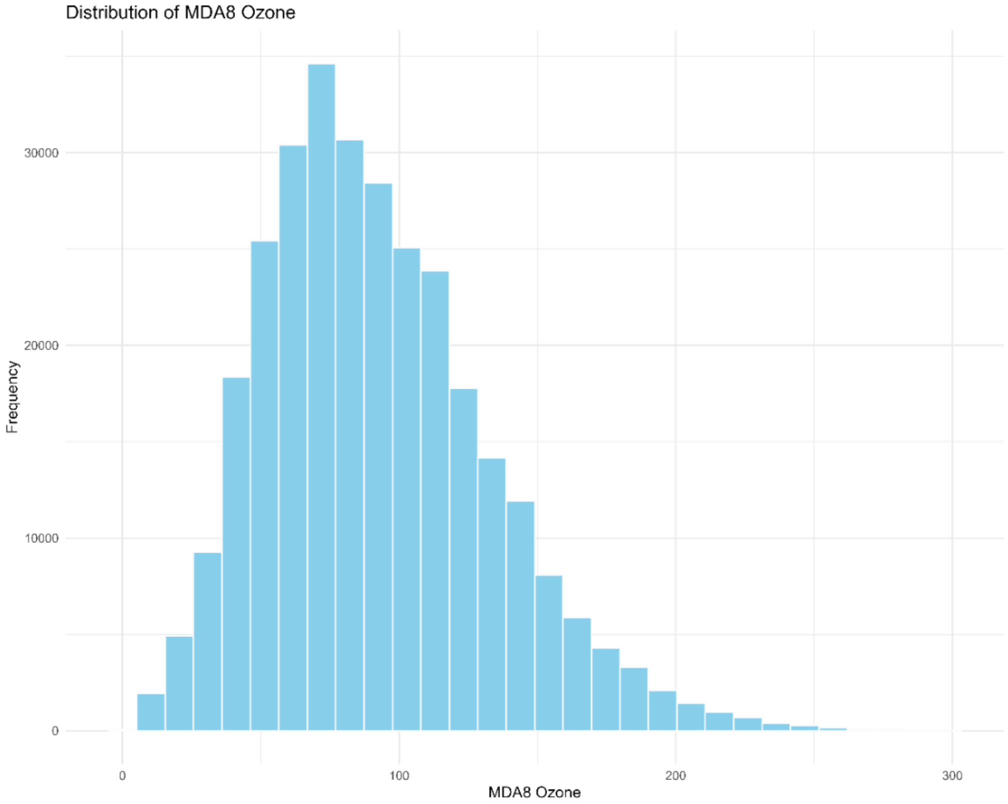

$$Y(\lambda) = \begin{cases} \frac{(Y^{\lambda} - 1)}{\lambda}, & if\ \lambda \neq 0 \\ \ln(Y), & if\ \lambda = 0 \end{cases} \quad \text{S1}$$

In addition, meteorological variables were standardized to account for differences in measurement units Eq.S2. The transformed O_3 was used as the response variable, and standardized meteorological data were included as covariates in the statistical models.[4–6]

$$X_{standardized} = \frac{X - \mu}{\sigma} \quad \text{S2}$$

Notably, Python was used for data merging and interpolation, and RStudio (Version 4.4) was used for data standardization and transformation, as well as for statistical inference.

Methods S2. Notes for Linear Mixed Models (LMMs).

LMMs provide a statistical framework appropriate for analysing the hierarchical data structures inherent to our study.[7,8] LMMs extend ordinary linear regression by incorporating both fixed effects and random effects. Fixed effects represent population-level effects, while random effects account for individual-specific deviations, allowing for the explanation of variability across different levels, such as subjects, locations, or time periods.[7] Mixed effects models have become a primary method for longitudinal data analysis and are often conceptualized as a two-stage analysis.[7,8]

In our context, the measurements of daily meteorological factors and O_3 concentrations are nested within provinces or monitoring sites. As such, the assumption of independent and identically distributed (i.i.d.) residuals is likely violated due to shared local meteorological, topographic, or emission conditions. The mixed effects model enables us to capture this dependence structure explicitly. Therefore, we employed an LMM to quantify the statistical association between extreme heat (EH) events and surface O_3 concentrations while accounting for potential heterogeneity of regions. The general form of the model is given by:

$$Y_{ij} = \beta_0 + \beta_1 D_{ij} + \beta X_{ij} + b_j + \epsilon_{ij} \quad \text{S3}$$

Where Y_{ij} is the MDA8 O_3 (O_3_8h_max) for day i in region j. D_{ij} is EH events (max_tem_35). X_{ij} is a vector of additional fixed effect covariates, including dew_tem, slp, wd, wsr, sctc and lpd. β is the corresponding vector of fixed effect coefficients. b_j is the random intercept for region j, capturing unobserved regional heterogeneity. ϵ_{ij} is the residual error term, assumed to be independent of b_j. The fixed effects estimate the average relationship across all regions, while the random effects account for deviations specific to each region. This hierarchical modelling structure improves statistical efficiency and yields more reliable standard errors in the presence of within-group correlation.

Notably, in within-provincial analysis, Beijing, Sichuan, Tianjin, and Ningxia each have only one site; thus, we used the linear model (LM) without random effects to explore the correlation in these areas.

Methods S3. Notes for Instrumental Variable (IV) Method.

The IV method corrects for bias caused by omitted variable confounding, simultaneity, or measurement error, which may lead to inconsistent estimates in the ordinary least squares (OLS) model.[9]

In our study, we hypothesize that EH may be endogenous due to unobserved meteorological or environmental factors that simultaneously influence O_3 formation. To address this concern, we used altitude as an external instrument. Altitude satisfies the relevance condition, as it is strongly correlated with temperature according to well-established atmospheric principles, whereby higher altitudes generally correspond to lower temperatures. It also satisfies the exogeneity condition, given that altitude, as a geographical attribute, is independent of unobserved economic or behavioural factors influencing the outcome variable. Its effect on the outcome is mediated solely through temperature, making it a suitable IV. Altitude has also been employed as an IV in prior research.[10–12]

The Two-Stage Least Squares (TSLS) model is the most commonly applied IV approach, addressing endogeneity issue. The model can be expressed as two stages. In the first stage, we estimated a predictive model for EH using altitude as an instrument and other exogenous controls, Eq.S4:

$$X_{ij} = \pi_0 + \pi IV_{ij} + \theta W_{ij} + u_{ij} \qquad \text{S4}$$

Where X_{ij} is EH for day i in site j. IV_{ij} is instrument variable (altitude). w_{ij} includes other exogenous covariates (dew_tem, slp, wd, wsr, sctc and lpd). u_{ij} is the error term. This step yields predicted values $\hat{X}_{ij}$, representing the component of EH variation attributable to exogenous variation in altitude.

In the second stage, we then modelled surface O_3 concentrations using the predicted values $\hat{X}_{ij}$ as the explanatory variable in an ordinary least square (OLS) model, Eq.S5:

$$Y_{ij} = \beta_0 + \delta\hat{X}_{ij} + \beta X_{ij} + \epsilon_{ij} \qquad \text{S5}$$

Here, Y_{ij} is the MDA8 O_3 concentration, X_{ij} includes other meteorological covariates. Similarly, ϵ_{ij} is the error term. The coefficient δ captures the causal effect of EH on O_3 levels, adjusted for endogeneity.

Methods S4. Notes for Heckman Two-step Selection (Heckman) Model.

This approach is designed to correct for selection bias in observational studies when the outcome is only observed or meaningful for a non-random subset of data.[13] In our study, the treatment group includes days that experience EH, while control days do not. To address potential selection bias due to the non-random occurrence of EH events, we applied the Heckman model. Since the occurrence of EH may correlate with unobserved factors that also influence O_3 levels, naive comparisons may lead to biased estimates. This method is particularly useful in our setting because EH days are not randomly distributed and may cluster in specific regions or seasons, where other unobserved factors also affect O_3 formation. By explicitly modelling the selection process, the

correction improves the validity of causal estimates under the assumption of a valid exclusion restriction that affects the probability of EH but not O_3 concentrations directly.

In the first stage, we estimated the probability of EH occurrence using a probit model Eq.S6:

$$D_i^* = Z_i\gamma + u_i \qquad \text{S6}$$

Here, D_i is a binary indicator for EH on day i, and Z_i is a vector of covariates that determine the likelihood of EH, including observation_field_meter, dew_tem, slp, wd, wsr, sctc, and lpd. The error term u_i follows a standard normal distribution. This model yields estimates of γ, which we used to compute the Inverse Mills Ratio (IMR):

$$\lambda_i = \frac{\phi(Z_i\hat{\gamma})}{\Phi(Z_i\hat{\gamma})} \qquad \text{S7}$$

where $\phi(\cdot)$ and $\Phi(\cdot)$ are the standard normal density and cumulative distribution functions, respectively. The IMR captures the conditional probability of being observed in the treated group and adjusts for non-random selection in the second stage.

In the second stage, we estimated the causal impact of EH on O_3 pollution using a LMM that includes the IMR as a covariate, Eq.S8:

$$Y_{ij} = \beta_0 + \delta D_{ij} + X_{ij}\beta + \rho\lambda_{ij} + b_j + \epsilon_{ij} \qquad \text{S8}$$

In this model, Y_{ij} is the $O_3\_8h\_max$ for day i in site j. D_{ij} is the treatment indicator for EH. X_{ij} is a vector of covariates including meteorological variables. λ_{ij} represents the IMR estimated from the first stage. b_j is a random intercept that accounts for unobserved heterogeneity across sites and ϵ_{ij} is the error term. The coefficient δ captures the adjusted causal effect of EH on O_3 concentrations. The term ρ indicates the extent of selection bias and tests whether correction is necessary. The inclusion of a random effect b_j controls for correlation within spatial clusters.

Methods S5. Notes for Propensity Scores (PS) Analysis.

Rosenbaum and Rubin[14] firstly introduced PS analysis to address imbalances in observed covariates between treatment and control groups in observational studies. The primary goal of PS analysis is to improve the comparability of these groups by adjusting for observed characteristics, thereby reducing confounding bias in effect estimates. The PS is defined as the conditional probability of receiving a specific treatment versus a comparator or no treatment, given the observed covariates X. It is expressed as:

$$e(X) = pr(Y = 1|X) \qquad \text{S9}$$

Where $e(X)$ is the PS function, $Y = 1$ represents observations in the treatment group, and $Y = 0$ represents observations in the control group. However, Rosenbaum and Rubin[14] emphasize that the comparability of treatment groups is conditional on the observed covariates included in the PS model and may not extend to unobserved factors. Therefore, balancing potential confounders through PS allows researchers to obtain a “quasi-randomization” of treatment groups, reducing confounding and improving effect estimation.[15] PS analysis relies on the Strong Ignorable Treatment Assignment (SITA) assumption, which includes two conditions: *unconfoundedness* and *positivity*.[14] The *unconfoundedness* assumption requires that all relevant pre-treatment characteristics are observed and included in the PS model, eliminating unmeasured confounding. The *positivity* assumption, also denoted as overlap or common support, requires that each observation has a non-zero probability of receiving any treatment value, mathematically expressed as Eq.S10:

$$0 < e(X) < 1 \qquad \text{S10}$$

This strategy is useful when treatment assignment, such as the occurrence of an EH event, is not randomized and may be influenced by multiple environmental or meteorological factors. Therefore, we employed PS analysis to estimate the causal effect of EH (exposure) on surface O_3 pollution (outcome). In our analysis, we calculated propensity scores as the probability of being exposed to EH. The scores were estimated via logistic regression, Eq.S11:

$$e(X_i) = P(\text{max\_}tem\text{\_}35_i = 1|X_i) = \frac{\exp(\beta_0 + \beta_1 X_{i1} + \beta_2 X_{i2} + \cdots + \beta_p X_{ip})}{1 + \exp(\beta_0 + \beta_1 X_{i1} + \beta_2 X_{i2} + \cdots + \beta_p X_{ip})} \quad \text{S11}$$

Where $max\_tem\_35_i$ is day i in EH (the treatment) group or not, and $e(X_i)$ is the probability that day i is EH day.

We applied three applications based on the estimated propensity score, including covariate adjustment (PSC), matching (PSM), and inverse probability of treatment weighting (IPTW). Notably, researchers generally begin by examining the distribution of propensity scores using histograms or density plots to ensure sufficient overlap between groups before matching.[16] Hence, we followed this order during the inference process. In all applications, we extended the estimation using LMM to account for within-region dependencies.

In PSC, the estimated propensity score is included as a covariate in the regression model. To account for clustered observations within sites, we use an LMM formation:

$$Y_{ij} = \beta_0 + \beta_1 D_{ij} + f(e(X_{ij})) + b_j + \epsilon_{ij} \quad \text{S12}$$

Where Y_{ij} is the O_3 level for observation i in site j. D_{ij} is the EH exposure. $e(X_{ij})$ is the estimated propensity score. b_j and ϵ_{ij} are random intercept and residual error, respectively. This model adjusts for both observed confounding (via the propensity score) and unobserved group-level heterogeneity (via random effects).

PSM is the most common PS application. By matching individuals with similar PS across treatment and control groups, the distribution of characteristics becomes more balanced, mitigating the influence of confounding factors on outcomes. Matching algorithms may be categorized as local or global, with nearest-neighbour (NN) matching as a common local algorithm. Under NN matching, each treated unit is paired with the closest control unit until all treated units find a match, thereby balancing characteristics between groups. In our study, daily observations are identified as individuals and are selected to match others. After matching treated and control observations based on their estimated propensity scores, we estimate the treatment effect:

$$Y_{ij} = \alpha + \tau D_{ij} + b_j + \epsilon_{ij} \quad \text{S13}$$

Here, matched pairs are used to reduce covariate imbalance. b_j and ϵ_{ij} are random intercept and residual error, respectively. The model estimates the average treatment effect on the treated (ATT). The ATT estimates how much O_3 concentrations would have decreased, on average, had EH events not occurred on the treated days. Covariate balance is assessed before and after matching. This application enables causal inference while controlling for spatial or temporal autocorrelation.

IPTW estimates the probability of treatment assignment based on observed covariates and assigns weights accordingly, enabling researchers to balance confounding variables such as meteorological factors distribution between treatment and control groups. By reweighting the treated and control observations based on the estimated propensity scores, IPTW helps create a pseudo-randomized scenario, thereby improving the validity of causal estimates.[17] In our context, for treated observations (EH days), they will be assigned weights equal to:

$$\frac{1}{PS} = \frac{1}{e(X_i)} \quad \text{S14}$$

And for controlled observations (non-EH days), they will be assigned weights equal to:

$$\frac{1}{(1 - PS)} = \frac{1}{1 - e(X_i)} \quad \text{S15}$$

The weighted LMM was then used to estimate the exposure effect while accounting for site-level heterogeneity, as shown in Eq.S13. The weights are applied to each observation. This application estimates the average treatment effect (ATE). The ATE estimates the impact of EH on O_3 concentrations if all observation days were randomly assigned to either EH or non-EH conditions.

Methods S6. Notes for Generalized Bootstrap (GB) Procedure.

To validate the robustness of the IPTW, we employed a procedure known as GB. Compared to traditional bootstrap methods, the approach developed by Li and Lawson[18] offers greater robustness and minimizes the risk of underestimated standard errors and inflated confidence levels. Specifically, traditional bootstrap methods rely on the multinomial distribution when applying it to propensity score analysis. Under this framework, the sampling probabilities assume that each subject has an equal likelihood of being selected, regardless of whether they belong to the treatment or control group. Consequently, the sampling probabilities are directly:

$$p_1 = p_2 = \cdots = p_n = \frac{1}{n_t}, q_1 = q_2 = \cdots = q_n = \frac{1}{n_c} \qquad \text{S16}$$

The GB procedure modifies the calculation of sampling probabilities, as outlined in Eq.S16, by incorporating individual propensity scores. This adjustment provides more stable and robust evidence for validating results.

In our study, the treatment group consists of observations during EH events, while the control group includes observations during non-EH days. By dividing the observations into treatment and control groups based on EH conditions, the sampling probabilities are separately controlled, thereby enhancing accuracy, particularly for propensity score analyses that require greater precision. The formula for calculating sampling probabilities in propensity score analysis is presented below. First, we compute the sampling probabilities as shown in Eq.S17.

$$p_i = \frac{\frac{1}{e_i}}{\sum_{i=1}^{n_t}\left(\frac{1}{e_i}\right)}, q_i = \frac{\frac{1}{(1-e_j)}}{\sum_{j=1}^{n_c}\left(\frac{1}{1-e_i}\right)} \qquad \text{S17}$$

where p_i is the sampling probability for treated units (observations during EH days), q_j is the sampling probability for control units, e_i is the propensity score of treated units i, and e_j is the propensity score of control units j.

After calculating this, we can draw sampling samples from each group in the multinomial distribution:

$$s_t \sim Multinominal\left(p_1, p_2, \dots, p_{n_t}\right) \qquad S18$$

$$s_c \sim Multinominal\left(q_1, q_2, \dots, q_{n_c}\right) \qquad \text{S19}$$

where s_t or s_c represents the numbers of times each treated (or control) subject appears in a bootstrap sample.

Focusing specifically on the IPTW, it is necessary to employ a properly weighted M-estimator that accounts for the heterogeneous sampling probabilities across individuals. The causal effect is estimated based on the GB samples s_t and s_c using the weighted least squares objective function:

$$min\left\{\sum_{i=1}^{n_t}\frac{k_i}{n_t p_i}f(\omega_i,\theta) + \sum_{j=1}^{n_c}\frac{k_j}{n_c q_j}f(\omega_j,\theta)\right\} \qquad \text{S20}$$

Here, ω_i denotes the cth covariates for treated observations during EH events, and k_i indicates the number of times observation i appears in the bootstrap sample s_t, drawn according to the GB procedure defined in Eq.S18 and Eq.S19. The function $f(\omega_i,\theta)$ represents the residual of the regression model for individual i. Analogously, the variables ω_j, k_j, and $f(\omega_j,\theta)$ correspond to the same quantities for control observations *j* during non-EH conditions from the sample s_c.

The regression parameters θ are estimated via weighted M-estimation in Eq.S20 in each new sample drawn by the GB procedure. This estimation is repeated over 1000 iterations of resampling from s_t and s_c under the GB scheme, ensuring comprehensive utilization of the data.

Methods S7. Notes for Transfer Entropy (TE) Method.

TE is an information-theoretic method that flexibly captures asymmetric and nonlinear dependencies between time series, without imposing strict distributional assumptions or requiring model parameter estimation.[19] This makes it suitable for complex environmental systems where feedbacks, delayed responses, and nonlinearity are prevalent. The core idea of TE is to quantify the information contribution of the source variable to the target variable by comparing the conditional probability distribution. TE has been applied to air pollution research, particularly to quantify the directional spillover effects of $PM_{2.5}$ concentrations between cities, and has demonstrated feasibility and strong potential in detecting spatiotemporal causality in environmental systems.[20,21]

By estimating the joint probability distribution and the conditional probability distribution, Shannon entropy and conditional entropy are calculated indirectly, thus achieving the calculation of TE. The corresponding mathematical formulation is presented as follows:

$$H(X|Y) = -\sum P(x,y)logP(x|y) \quad \text{S22}$$

where $H(X|Y)$ is the conditional entropy of a random variable X given a random variable Y. $P(x,y)$ is the joint probability distribution of random variable X and Y. $P(x|y)$ is the conditional probability of X given that $(Y = y)$.

In our study, TE was used to explore the temporal relationship and to infer the causal direction between EH and surface O_3 pollution. The source variable (X) is EH, and the target variable (Y) is O_3. We depend on the difference between the conditional entropy of EH and O_3 to differentiate their sequences of occurrence. Here is the formula in terms of the temporal sequence:

$$T_{X\to Y} = H(y_{t+1}|y_t^L) - H(y_{t+1}|y_t^L, x_t^K) \quad \text{S23}$$

Where $y_t^L = \{y_t, y_{t-1}, \dots, y_{t-L+1}\}$ denotes the past L values of Y, $x_t^K = \{x_t, x_{t-1}, \dots, x_{t-K+1}\}$ denotes the past K values of X. $H(y_{t+1}|y_t^L)$ represents the uncertainty in predicting y_{t+1} using only the past information of Y. $H(y_{t+1}|y_t^L, x_t^K)$ represent the uncertainty in predicting y_{t+1} using both the past information of Y and X. If $H(y_{t+1}|y_t^L, x_t^K)$ is significantly smaller than $H(y_{t+1}|y_t^L)$, then $T_{X\to Y} > 0$, indicating that X provides additional predictive information about Y.

The selection of the lag parameters is based on both scientific mechanisms and empirical data. Specifically, the time delay for EH was set to $L_x = 2$, considering that O_3 formation responds to heat with a short delay due to cumulative photochemical processes such as peroxyacetylnitrate (PAN) decomposition and volatile organic compounds (VOCs) oxidation.[22] Previous findings also suggest that O_3 peaks may lag behind EH by 1-2 days due to the inhibition of isoprene emissions under extremely high temperatures.[23,24] Meanwhile, the time delay for O_3 itself was set to $L_y = 1$, reflecting its short-lived autocorrelation structure caused by rapid diurnal fluctuations, i.e., nighttime NO titration and daytime photochemical generation.[25] Finally, the number of guided samples was set to 100, which was used to calculate the p-value to assess the significance of the TE.

Supplemental references

1. A. Smith, J., and E. Todd, P. (2005). Does matching overcome LaLonde's critique of nonexperimental estimators? J. Econometrics *125*, 305–353. https://doi.org/10.1016/j.jeconom.2004.04.011.

2. Caliendo, M., and Kopeinig, S. (2008). Some practical guidance for the implementation of propensity score matching. J. Econ. Surv. *22*, 31–72. https://doi.org/10.1111/j.1467-6419.2007.00527.x.

3. Noor, M.N., Yahaya, A.S., Ramli, N.A., and Al Bakri, A.M.M. (2013). Filling missing data using interpolation methods: Study on the effect of fitting distribution. Key Eng. Mater. *594–595*, 889–895. https://doi.org/10.4028/www.scientific.net/KEM.594-595.889.

4. Eastoe, E.F. (2009). A hierarchical model for non-stationary multivariate extremes: A case study of surface-level ozone and NOx data in the UK. Environmetrics *20*, 428–444.

https://doi.org/10.1002/env.938.

5. Gal, M., and Rubinfeld, D.L. (2018). Data standardization. SSRN Electron J. https://doi.org/10.2139/ssrn.3326377.

6. Graf-Jaccottet, M. (1993). A flexible model for ground ozone concentration. Environmetrics *4*, 23–37. https://doi.org/10.1002/env.3170040103.

7. Cheng, J., Edwards, L.J., Maldonado ‐ Molina, M.M., Komro, K.A., and Muller, K.E. (2010). Real longitudinal data analysis for real people: Building a good enough mixed model. Stat. Med. *29*, 504–520. https://doi.org/10.1002/sim.3775.

8. Laird, N.M., and Ware, J.H. (1982). Random-effects models for longitudinal data. Biometrics *38*, 963–974. https://doi.org/10.2307/2529876.

9. Hogan, J.W., and Lancaster, T. (2004). Instrumental variables and inverse probability weighting for causal inference from longitudinal observational studies. Stat. Methods Med. Res. *13*, 17–48. https://doi.org/10.1191/0962280204sm351ra.

10. Barreca, A.I. (2012). Climate change, humidity, and mortality in the United States. J. Environ. Econ. Manage. *63*, 19–34. https://doi.org/10.1016/j.jeem.2011.07.004.

11. Burke, M., Hsiang, S.M., and Miguel, E. (2015). Global non-linear effect of temperature on economic production. Nature *527*, 235–239. https://doi.org/10.1038/nature15725.

12. Graff Zivin, J., and Neidell, M. (2014). Temperature and the allocation of time: Implications for climate change. J. Labor Econ. *32*, 1–26. https://doi.org/10.1086/671766.

13. Heckman, J. (1978). Dummy endogenous variables in a simultaneous equation system. Econometrica *46*, 931–959. https://doi.org/10.2307/1909757.

14. Rosenbaum, P.R., and Rubin, D.B. (1983). The central role of the propensity score in observational studies for causal effects. Biometrika *70*, 41–55. https://doi.org/10.1093/biomet/70.1.41.

15. Ali, M.S., Prieto-Alhambra, D., Lopes, L.C., Ramos, D., Bispo, N., Ichihara, M.Y., Pescarini, J.M., Williamson, E., Fiaccone, R.L., Barreto, M.L., et al. (2019). Propensity Score Methods in Health Technology Assessment: Principles, Extended Applications, and Recent Advances. Front. Pharmacol. *10*, 973. https://doi.org/10.3389/fphar.2019.00973.

16. Dehejia, R.H., and Wahba, S. (2002). Propensity score-matching methods for nonexperimental causal studies. Review of Economics and Statistics *84*, 151–161. https://doi.org/10.1162/003465302317331982.

17. Austin, P.C. (2011). An introduction to propensity score methods for reducing the effects of confounding in observational studies. Multivariate Behav. Res. *46*, 399–424. https://doi.org/10.1080/00273171.2011.568786.

18. Li, T., and Lawson, J. (2024). A generalized bootstrap procedure of the standard error and confidence interval estimation for inverse probability of treatment weighting. Multivariate Behav. Res. *59*, 251–265. https://doi.org/10.1080/00273171.2023.2254541.

19. Schreiber, T. (2000). Measuring information transfer. Phys. Rev. Lett. *85*, 461–464. https://doi.org/10.1103/PhysRevLett.85.461.

20. Hu, H., Tan, Z., Liu, C., Wang, Z., Cai, X., Wang, X., Ye, Z., and Zheng, S. (2022). Multi-timescale analysis of air pollution spreaders in Chinese cities based on a transfer entropy network. Front. Environ. Sci. *10*, 970267. https://doi.org/10.3389/fenvs.2022.970267.

21. Seong, N. (2021). Deep spatiotemporal attention network for fine particle matter 2.5

concentration prediction with causality analysis. IEEE Access *9*, 73230–73239. https://doi.org/10.1109/ACCESS.2021.3080828.

22. Steiner, A.L., Davis, A.J., Sillman, S., Owen, R.C., Michalak, A.M., and Fiore, A.M. (2010). Observed suppression of ozone formation at extremely high temperatures due to chemical and biophysical feedbacks. Proc. Natl. Acad. Sci. U.S.A. *107*, 19685–19690. https://doi.org/10.1073/pnas.1008336107.

23. Sillman, S., and Samson, P.J. (1995). Impact of temperature on oxidant photochemistry in urban, polluted rural and remote environments. J. Geophys. Res. Atmos. *100*, 11497–11508. https://doi.org/10.1029/94JD02146.

24. Steiner, A.L., Tonse, S., Cohen, R.C., Goldstein, A.H., and Harley, R.A. (2006). Influence of future climate and emissions on regional air quality in california. J. Geophys. Res. Atmos. *111*, 2005JD006935. https://doi.org/10.1029/2005JD006935.

25. Xie, Q., Tham, Y.J., Yu, X., Wang, Z., Ling, Z., Wang, X., Guo, H., and Wang, T. (2023). Seasonal variations of O3 formation mechanism and atmospheric photochemical reactivity during severe high O3 pollution episodes in the pearl river delta region. Atmos. Environ. *309*, 119918. https://doi.org/10.1016/j.atmosenv.2023.119918.
